# Supplementary figures and images for: In vivo dynamics and adaptation of HTLV-1-infected clones under different clinical conditions
Source: PLoS Pathog. 2021 Feb 1;17(2):e1009271. doi: 10.1371/journal.ppat.1009271 (PMC7877780; doi:10.1371/journal.ppat.1009271)

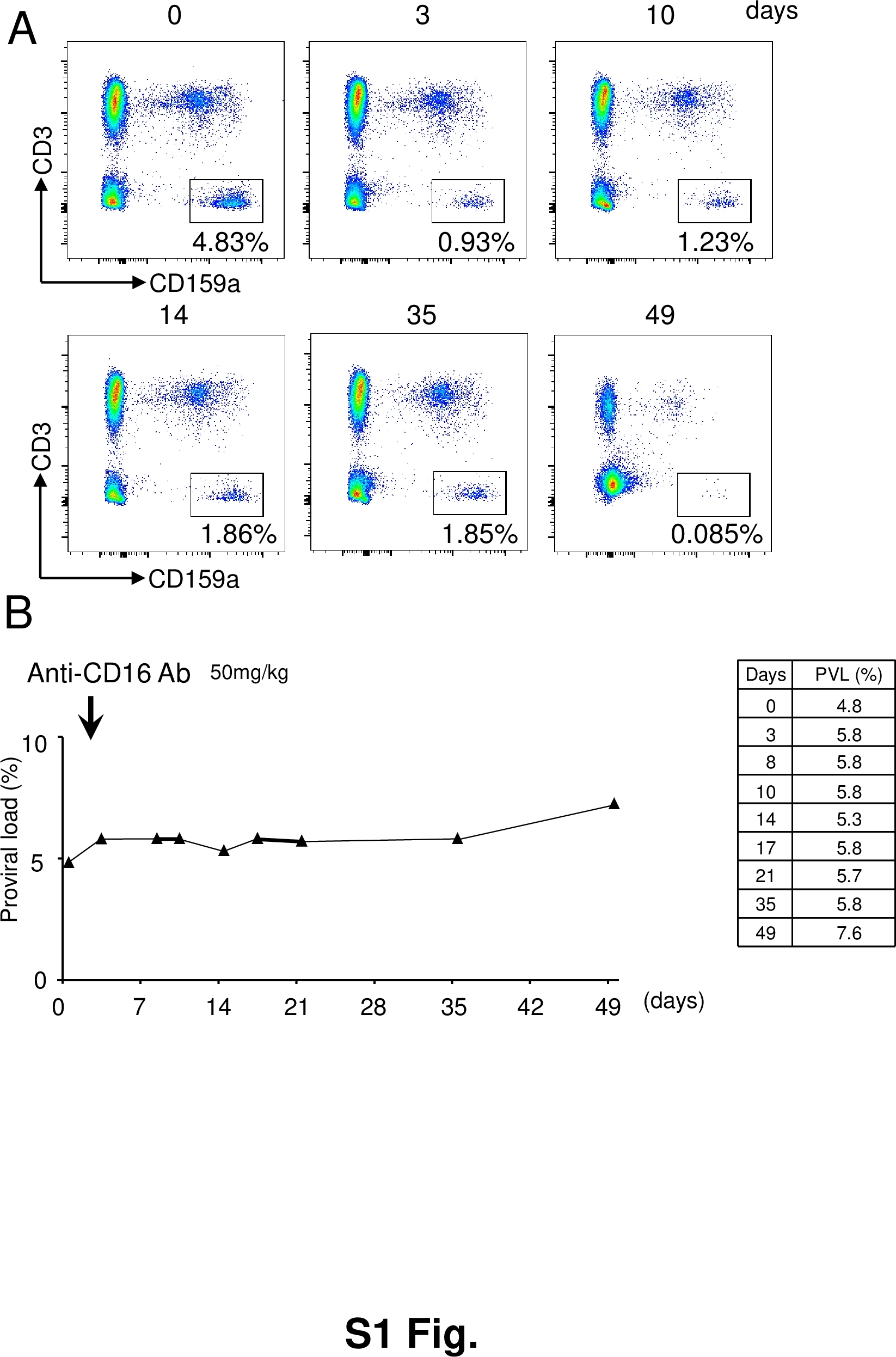

Supplement: S1 Fig — Anti-CD16 antibody was administered into a STLV-1 infected Japanese macaque. (A) To avoid blocking of CD16 detection by the administered antibody, NK cells (CD3-CD159A+ lymphocytes) were detected before and after administration of anti-CD16 antibody. (B) Proviral load of PBMCs is shown. The time of administration of antibody is indicated by the arrows. (TIF) [file ppat.1009271.s001.tif]
