# Supplementary material for: In vivo dynamics and adaptation of HTLV-1-infected clones under different clinical conditions
Source: PLoS Pathog. 2021 Feb 1;17(2):e1009271. doi: 10.1371/journal.ppat.1009271 (PMC7877780; doi:10.1371/journal.ppat.1009271)
Supplement: Attachment — Submitted filename: Rebuttals.docx [file ppat.1009271.s006.docx]

We are grateful to reviewers for the critical comments and valuable suggestions that have helped us improve our paper. As indicated in the following responses, we have taken all these comments and suggestions into account in the revised version.

Major issues

Reviewer #2:

Comment 1: In Fig. 1, the immune-suppressive treatments (Fig. 1A top, mycophenolate, prednisolone, tacrolimus) presumably suppressed the recipient’s CTL response since the transplanted liver seemed tolerated up to 15 months. If so, the HTLV-1-infected T cells from the donor should have been immune tolerated as well. As such, it is erroneous and misleading to conclude that HTLV-1-infected clones were turning over in the transplant recipient because of host immune surveillance (lines 111-116). For this conclusion/ suggestion to be drawn/made, a direct demonstration of increased anti-HTLV-1 CTL response in the transplant recipient is needed. However, in view of the immunosuppressive treatments, such evidence is unlikely to be found. A more likely scenario is outlined below in comment 2 for the authors to consider.

Response: We appreciate this valuable comment on our study. This patient was treated with immunosuppressive agents for transplantation. However, this patient was quickly seroconverted, indicating that immune system of this patient responded to HTLV-1. Further, we detected IFNγ production to Tax peptides in this patient using ELISPOT assay (Table 1) in the presence of immunosuppressive agents. The number of IFN-γ spots responding to Tax peptides are similar to those observed in HAM/TSP patients and a HTLV-1 carrier. This is because these immunosuppressive agents partially suppress the host immune responses. This data shows T-cell responses to Tax in this patient. We described these findings in the text (page 6, line 106-108).

Comment 2: The most likely interpretation of the data in Fig. 1 is that the cytopathic effects of HTLV-1, not immune surveillance, caused the death of most newly infected T cells by inducing cell cycle arrest, senescence or apoptosis as has been extensively documented in the literature (which should be cited, by the way). This then led to the rapid turn-over of the infected cell population initially, and selected for latently infected cell clones that expressed viral antigens at low levels or intermittently.

Response: We appreciate this valuable comment from Reviewer 2. We agree that HTLV-1 induced cytopathic effects induce senescence/cell death of newly infected cells. Since the cell number of several clones at one month was more than ten, these clones proliferate at one month. As Reviewer suggests, it is possible that intermittent Tax expression induces senescence as previously reported (Kuo YL and Giam CZ, EMBO J, 2006), and activation-induced cell death through upregulated Fas ligand expression (Rivera I, et al., JBC, 10998). We described these in page 12 line 250-page 13 line 261. T-cell responses to Tax peptides and antibody to HTLV-1 were detected in this patient. This data shows that immune surveillance is also implicated in selection of HTLV-1 infected clones.

Comment 3: For Fig. 2, the rationale and experimental details for the HSCT study are not clear, making the interpretation of the results difficult to understand. What were the conditioning regimens used for the HSCT recipients? Was the HSCT allogeneic? Were bone marrows or CD34+ stem cells used in the transplantation? Clinical protocols/treatments should be described and substantively discussed for proper understanding and interpretation of the data.

Response: We added the information of HSCT as Table 2.

Comment 4: In Fig. 2, were infected CD4+ T cells or stem cells transplanted into recipients? What were the PVLs in the transplants? What were the numbers of susceptible CD4+ cells throughout the study? Were the hematopoietic systems fully reconstituted after the transplantation? The newly reconstituted hematopoietic systems in the HSCT recipients were expected to reflect those of the donors. As such, it is perhaps not surprising that the HTLV-1-infected clones in the recipients were mostly donor-derived. The authors may want to address whether these clones derived from donor T cells or donor stem cells, and to what extent were donor T cells transplanted during HSCT?

Response: Since we did not isolate CD34+ cells, infused cells contained infected CD4+ T cells. Clinical data of these patients are shown in Table 2. During transplantation, the immune system of the recipients is strongly affected by allogeneic transplantation and immunosuppressive agents. We speculate that these factors allow *de novo* infection or influence the clonality of HTLV-1 infected cells. However, HTLV-1 infected clones are stably present in the recipients.

Comment 5: For the JM experiment, it would be helpful to know when and how the JM under study was infected and for how long.

Response: These JMs were maintained in the colony of Primate Research Institute of Kyoto University. It is likely that STLV-1 transmits via breast-feeding and sexual intercourse. However, we did not identify how these JMs in this study are infected.

Comment 6: The outcome of HTLV-1 (or HIV, HSV, etc.) infection is a balance between immune surveillance and the intrinsic pathogenicity of HTLV-1 coupled with its ability to integrate and undergo selection. The cytopathic effect of HTLV-1 was completely ignored in this paper (Lines 137-139).

Response: We appreciate these valuable comments (comment 6-8). Indeed, transient Tax expression causes senescence and suppress cell cycling. Furthermore, Tax expression induces Fas ligand expression, resulting in activation-induced cell death of T cells. In addition to effects of host immune responses, we discussed about cytopathic effects of HTLV-1 in in page 12 line 248-page 13 line 259.

Comment 7: The increased turn-over of infected clones after CD8 depletion in JM suggests that there was continual viral reactivation and de novo infection in STLV-infected JM, and many of the infected cells turned over not only because of CTL killing (which was suppressed by the antibody), but most likely because of the cytopathicity caused by the replication. Here again, the reduction in stax mRNA/STLV proviral DNA after CD8 depletion (Fig. 4A right panel) is consistent with the cytopathic effects of the infection, which likely causes DNA damage, cell cycle arrest, senescence, and apoptosis in cells undergoing viral replication, thus leading to the survival of cells that are not replicating HTLV-1 (lower stax/PVL). As mentioned in comment 2, this has been shown by others and should be discussed in the present context with proper citations.

Response: We agree that cytopathic effects of HTLV-1 might be involved in changed clonality after depletion of CD8+ T cells in STLV-1 infected JMs. We described this in page 14 line 288-295.

Comment 8: In the discussion (lines 289-301), the infected ATL clones that express Tax intermittently are interesting, but these clones had been selected in vivo during chronic infection. The data presented in the study seem more consistent with the notion that the majority of HTLV-1-infected cells turned over rapidly even in the absence of immune control, mostly due to the cytotoxic nature of the infection. Again, this point should be discussed.

Response: We described about cytopathic effects of HTLV-1 as described above. In this section, we described about transient Tax expression in ATL cases. Data from depletion of CD8+ T cells in JMs is regarding STLV-1 infected cells.

Minor issues

Reviewer 1

Comment 1: Fig 3_ Does the anti-CD8 antibody used for the flow detection bind to the CD8 T-cells despite the presence of the administered CD8 antibody? If they compete for the epitope or cause steric hindrance to each other, then the flow detection should combine/compare indirect staining of the cells bound by the administered CD8 antibody (by using Fluorophore-labeled anti-simian IgG) and direct staining. Though chances are slim that CD8 T-cell depletion did not occur in vivo, that could change the interpretation, if true.
This goes with the CD16 depletion assay.
Alternatively, if CD8 T-cells did not decrease in number, but only their presence was “stealthed”, that should lead to the increase of CD4CD8 double negative cells. Thus, if the ratio of CD4 vs. nonCD4/CD8 did not show overt decrease in the early days of treatment, it would strongly suggest that the observation supports the specific depletion of CD8 T-cells by the antibody treatment.

Response: We thank valuable comments from Reviewer 1. Another report used the same combination of anti-CD8 antibody (M-T807R1 for depletion of CD8+ T cells and RPA-T8 for detection of CD8+ T cells)(Watkins JD, et al., PLoS One, 6: e18207, 2011). Therefore, M-T807R1 indeed depleted CD8+ T cells. For NK cells, we corrected the data of NK cells. We measured CD3^-^CD159A^+^ lymphocytes as NK cells to avoid blocking of CD16 detection by the administered antibody (page 21, line 450-452), and presented this data in supplementary Figure 1.

Comment 2: Page 27 line 470 _ digital detection of “HTLV-1 type 2 provirus” the word type 2 provirus is misleading as it refers to ” the type 2 defective mutants”, but not a new type of HTLV-1.

Response: We changed this according to Reviewer’s suggestion.
