# Supplementary material for: In vivo dynamics and adaptation of HTLV-1-infected clones under different clinical conditions
Source: PLoS Pathog. 2021 Feb 1;17(2):e1009271. doi: 10.1371/journal.ppat.1009271 (PMC7877780; doi:10.1371/journal.ppat.1009271)
Supplement: Attachment — Submitted filename: Rebuttals.docx [file ppat.1009271.s007.docx]

We thank valuable comments and suggestions from Reviewer 2.

Comment 1. The statement in page 6, lines 116-119 of the revised manuscript ⎯ “Thus, newly-infected HTLV-1 clones dynamically changed, suggesting that appropriate HTLV-1-infected clones for survival in vivo are selected under host immune surveillance. It is noteworthy that many donor-derived HTLV-1 cells persisted in the recipient (Fig 1D).” ⎯ remains difficult to reconcile and understand. How can the immune surveillance, on the one hand, controlled the newly infected clones in the transplant recipient, and yet leaving donor-derived HTLV-1-infected cells to persist untouched? This point was raised in reviewer 2, comments 2 and 4 of the previous review.
The manuscript can be improved by considering the following possibilities: (i) The newly infected cells turned over rapidly due to the cytopathic effect of HTLV-1, not primarily due to the host immune response, which would have eliminated both newly infected cells and donor HTLV-1 cells. (ii) Alternatively, only a very small fraction of the previously selected donor HTLV-1 cells were capable of virus replication and transmission (through sporadic reactivation?), and as such most of them evade immune detection.

Response: We deleted “under host immune surveillance” in line 118 of page 6. It is possible that HTLV-1-infected cells are selected by host immune responses and cytopathic effects of viral proteins. The clones with appropriate level of viral gene expression are selected by host immune responses and cytopathic effects since infected cells with overexpression of Tax are likely eliminated by cytotoxic T cells and cytopathic effects of Tax. Donor infected cell persisted *in vivo* in contrast to newly infected cells as shown in Figure 1D. These cells are already selected by host immune responses and cytopathic effects of viral proteins. HTLV-1-infected cells with appropriate expression of viral genes (escape from host immune surveillance and cytopathic effects) persist in vivo. It is very difficult to differentiate effects of the host immune surveillance and cytopathic effects. Therefore, we described the possibility of both factors in this manuscript.

I would like to mention the following findings. After seroconversion, we detected clonal proliferation of some clones at one month (Figure 1C), which means that these clones proliferate without strong cytopathic effects. However, these clones were suppressed or disappeared later, suggesting that the host immune response are implicated in this dynamic changes.

Comment 2: 2. The following literature should also be cited and discussed:
(i) Glowacka et al “Delayed Seroconversion and Rapid Onset of Lymphoproliferative Disease After Transmission of Human T-Cell Lymphotropic Virus Type 1 From a Multiorgan Donor”
(<https://academic.oup.com/cid/article/57/10/1417/289550>)
(ii) Cook et al “Rapid dissemination of human T-lymphotropic virus type 1 during primary infection in transplant recipients”
Both papers suggest that the host immune response to transplant-associated HTLV-1 infection, while present, is not very robust.
(<https://retrovirology.biomedcentral.com/articles/10.1186/s12977-015-0236-7>).
(iii) An earlier study had shown that the majority of cells newly infected by HTLV-1 in cell culture became arrested in cell cycle progression or senescent, and only a small fraction of cells that expressed Tax at low levels managed to undergo expansion. (<https://pubmed.ncbi.nlm.nih.gov/24699669/>)

Response: Patients reported in Retrovirology (Cook et al.,) were seroconverted just after transplantation from the seropositive donor as well as the patient of this study. Importantly, the report in Retrovirology did not analyze the immune response. We quoted this reference in page 12. In addition, we quoted the paper by Phillip et al., in page 13.

The case reported by Glowacka et al. is very interesting, which suggests delayed seroconversion in these transplanted patients. However, seroconversion is quickly observed in a transplanted patient from the seropositive donor (Kikuchi H et al., BMT, 2000) as well as the paper (Cook et al., Retrovirology) and our study. These indicates that the patients are seroconverted just after transplantation.
